# Targeting Inflammation in Non-Small Cell Lung Cancer through Drug Repurposing

**DOI:** 10.3390/ph16030451

**Published:** 2023-03-16

**Authors:** Thiviyadarshini Rajasegaran, Chee Wun How, Anoosha Saud, Azhar Ali, Jonathan Chee Woei Lim

**Affiliations:** 1 Department of Medicine, Faculty of Medicine and Health Sciences, Universiti Putra Malaysia, Serdang 43400, Selangor, Malaysia; 2 School of Pharmacy, Monash University Malaysia, Bandar Sunway, Subang Jaya 47500, Selangor, Malaysia; 3 Cancer Science Institute Singapore, National University of Singapore, Singapore 117599, Singapore

**Keywords:** non-small cell lung cancer, inflammation, drug-repurposing, inhalation, drug delivery

## Abstract

Lung cancer is the most common cause of cancer-related deaths. Lung cancers can be classified as small-cell (SCLC) or non-small cell (NSCLC). About 84% of all lung cancers are NSCLC and about 16% are SCLC. For the past few years, there have been a lot of new advances in the management of NSCLC in terms of screening, diagnosis and treatment. Unfortunately, most of the NSCLCs are resistant to current treatments and eventually progress to advanced stages. In this perspective, we discuss some of the drugs that can be repurposed to specifically target the inflammatory pathway of NSCLC utilizing its well-defined inflammatory tumor microenvironment. Continuous inflammatory conditions are responsible to induce DNA damage and enhance cell division rate in lung tissues. There are existing anti-inflammatory drugs which were found suitable for repurposing in non-small cell lung carcinoma (NSCLC) treatment and drug modification for delivery via inhalation. Repurposing anti-inflammatory drugs and their delivery through the airway is a promising strategy to treat NSCLC. In this review, suitable drug candidates that can be repurposed to treat inflammation-mediated NSCLC will be comprehensively discussed together with their administration via inhalation from physico-chemical and nanocarrier perspectives.

## 1. Introduction

According to GLOBOCAN 2020, lung cancer tops the cancer list, with highest fatality rate and accounts for 18% of all cancer deaths worldwide [1]. About 84% of lung cancer cases belongs to non-small cell lung carcinoma (NSCLC) and the remaining 15% belongs to small cell lung carcinoma (SCLC) [2]. NSCLC is categorized into three sub-types: adenocarcinoma, squamous cell carcinoma, and large cell carcinoma. Adenocarcinoma is the major subtype with about 45% of all NSCLC followed by squamous cell carcinoma with 25–30% and the remaining 5–10% is large cell carcinoma subtype [3]. Late diagnosis of disease (at stage III and IV) is the major factor for the poor survival rate in lung cancer patients as the disease has progressed to the metastatic stage. About 92% of patients diagnosed at stage IA1 could survived for 5 years or more compared to 10% of patients diagnosed at stage IV. Furthermore, slight enlargement in tumor size from <1 cm (stage IA1) to >2 cm (stage IA3) could reduce the 5-year survival rate of patients from 92% to 77% [4].

Current NCSLC therapies, including surgery, chemotherapy, and radiotherapy, are insufficient to reduce the high mortality rates. These approaches lack precision and are usually limited by low drug bioavailability due to high first pass metabolism. Furthermore, serious adverse effects occur due to non-specificity where the chemotherapeutics adversely affect healthy cells [5]. To improve the survival of NSCLC patients, personalized medicine is preferred. Recent molecular targeted therapies, such as epidermal growth factor receptor tyrosine kinase inhibitors (EGFR-TKIs), could restrict growth and proliferation of lung tumors with EGFR mutations. On the other hand, targeting ROS could inhibit signaling pathways, such as MAPK/ERK, JAK/STAT, and P13K/AKT/mTOR. Then, targeting BRAF can interfere in cell proliferation and growth [6]. However, this treatment is only effective for a short duration due to subsequent development of acquired drug resistance [7]. Anaplastic lymphoma kinase (ALK) mutation is another example of a successful targeted therapy approach. Crizotinib is an FDA-approved agent that targets tyrosine kinases, such as ALK, c-mesenchymal-epithelial transition (c-MET), and c-ros oncogene 1 (ROS). Crizotinib has shown promising improvement in progression-free survival, and was the first ALK-tyrosine kinase inhibitor approved in the treatment of ALK-rearranged NSCLC [8]. However, treatment with Lorlatinib have indicated a survival rate of only 12 out 37 patients who were ALK positive, while 8 out of 14 patients who were positive for ROS survived [9].

Another targeted approach, immunotherapy, is also currently being applied in NSCLC treatment. Anti-PD-1/PDL-1 inhibitors, such as nivolumab and Atezolizumab, have shown substantive clinical activity in metastatic lung cancer and are approved for first or subsequent lines of therapy. However, only 20% of NSCLC patients showed significant remission and clinical benefit due to development of resistance [10]. Dysregulation of immune balance was found involved in promoting the progression of cancer. Tumor cell vaccines and antigen-specific vaccines have been suggested to shift immune balance in favor of host and elicit an immune response against the antigens. Belagenpumatucel-L is an allogenic tumor vaccine comprising a pool of four irradiated transforming-growth factor (TGF)-β-modified NSCLC lines. A group of randomized patients who had earlier completed chemotherapy within 12 weeks were treated with this vaccine and showed improvement in overall survival by 21 months when compared to the placebo group, which showed improvement of overall survival by 14 months [3]. The goal of these therapies, nevertheless, aims to lengthen progression-free survival instead of prevention. Lifestyle and environmental factors are well known to be tightly associated with lung cancer. Cigarette smoking accounts for 85–90% of lung cancer, depending on the extent of smoking and exposure to other carcinogenic factors such as asbestos. Other risk factors include ionizing radiation, radon, environmental toxins, metals (arsenic, chromium and nickel), history of pulmonary fibrosis, HIV infection, and alcohol consumption [11].

Among other factors, inflammation plays an integral role in initiating and supporting tumor growth. Physiologically, inflammation is an important process where the body responds to stimuli such as irritants, pathogens, injuries and eliminates tissue and cell damage. Nevertheless, unregulated inflammation can induce organ fibrosis and cancer [12]. Tumor inflammation is now recognized as one of the “10 characteristics of cancer”. Inflammation is thought to enhance tumor progression and development by supplying pro-tumorigenic constituents to tumor microenvironment [13]. It also contributes at all stages of tumorigenesis, from malignant transformation and tumor initiation to invasion and metastasis [14]. Here, we review the impact of inflammation-mediated mechanisms in the development and progression of NSCLC, and identify potential targets for therapeutic intervention. We will also examine current drugs and natural compounds with anti-inflammatory properties for drug repurposing, and the delivery of repurposed drugs by inhalation to improve treatment efficacy.

## 2. Inflammation in NSCLC Initiation and Progression

Lung cancer often occurs with a well-defined inflammatory tumor microenvironment. The tumor microenvironment (TME) is formed by various events known as tumorigenesis, progression, invasion, and metastasis [14]. The TME is highly complex, comprising cytokines, vasculature, growth factorsof different populations of stromal cells, such as tumor-associated macrophages (TAMs), tumor-associated fibroblasts (TAFs), and myeloid-derived suppressor cells (MDSCs) [15]. Continuous inflammatory conditions can induce DNA damage and mutations and increased cell division rate damage to lung tissue. Chronic infection, physical inactivity, diet, (visceral) obesity, social isolation, intestinal dysbiosis, sleep disruption, mental stress, and circadian rhythm disruption as well as exposure to xenobiotics such as air pollutants, oxidants, hazardous waste products, gases, poisons, smoking, and lung diseases, have all been found to elevate the risk of developing inflammation in the lung [16,17]. Inflammation can either initiate or promote anti-apoptotic signals and elevate risk of lung cancer. Additionally, inflammation promotes angiogenesis or growth of new blood vessels to provide nutrients to tumor cells [14].

It requires long-term exposure to these factors to induce cancer via inflammation. In a recent clinical study involving 311 NSCLC patients, inflammation was utilized as a prognostic marker for treatment outcome after receiving first-line chemotherapy or targeted therapy for advanced NSCLC. The Systemic Immune-Inflammation Index (SII), which was derived by analyzing neutrophils, platelet counts, and lymphocytes from peripheral patient blood samples, was designed and utilized to measure the impact of inflammation towards cancer progression. Out of 311 patients, 179 were categorized into group A, with SII ≥ 1270, while the remaining patients were categorized into group B, with SII < 1270. The median overall survival was 12.4 months for patients in Group A and 21.7 months for patients in Group B. Median progression free survival was 3.3 and 5.2 months, respectively for both groups. The study concluded that inflammation played a major impact in the prognostic outcome of NSCLC. Furthermore, SII was highly recommended to be included as a tool to measure prognostic impact of patients and this tool applies not only for locally advanced but also for metastatic NSCLC [18]. A meta-analysis study on the prognostic value of pre-treatment advanced lung cancer inflammation (ALI) index in NSCLC was recently conducted. ALI is a tool used to access systemic inflammation in patients by assessing their BMI, serum albumin, and neutrophil-to-lymphocyte ratio. A combination of 14 studies involving 3607 patients concluded that pretreatment ALI was found to be a reliable prognostic marker for NSCLC at both early and late stage. Lower pretreatment ALI indicates poor overall survival and progression free survival [19]. Comparing SII and pretreatment ALI, SII has more inflammatory background compared to pretreatment ALI, as the pretreatment ALI correlated with the immune-nutritional status of the patients.

Lung tumor progression occurs as a result of interaction between intrinsic (genetic) and extrinsic (environment) pathways. Intrinsic pathway is driven strongly by genetic events, such as oncogenes and genetic aberrations, leading to neoplastic transformation and also initiates the construction of an inflammatory microenvironment [20]. In the extrinsic pathway, the inflammatory condition is mainly developed by inflammatory leukocytes, particularly macrophages and soluble mediators such as histamine, serotonin, bradykinin, and other vasoactive amines including eicosanoids, such as prostaglandins, leukotrienes, and thromboxanes [21].

Abnormal inflammation can be induced by oncogene activation and/or silencing of tumor suppressor genes. Genetic alterations in EGFR and KRAS are commonly found in human lung adenocarcinoma. EGFR mutations are more frequent among non-smokers, whereas KRAS mutations are more common in tobacco smokers [12]. KRAS mutation causes elevated secretion of VEGF and CXCL1, which support cancer progression and development [22]. COPD-like inflammation was recently reported to promote lung cancer in KRAS mutant mouse model. Both HIF-1 and HIF-1α activities were found significantly elevated in lung tumors of KRAS mutant mice. Regardless of either absence or presence of COPD, the number of lung tumor surface was found to be significantly reduced in HIF-1α deficient mice. Angiogenesis and cell proliferation activities were also found to be reduced. However, these observations were seen only with HIF-1α expression, indicating a key target for lung cancer progression [23]. HIF-1α is a transcription factor and modulates cellular response to low oxygen through orchestrating a metabolic switch to allow cell survival. Inflamed tissues are often hypoxic, and HIF-α allows immune cells such as macrophages, dendritic cells, neutrophils, T cells, and B cells to adapt by regulating cellular metabolism and expression of immune-related genes to suppress activity of immune cells in early stages of tumor development [24]. Under hypoxic condition, HIF-1α was highly expressed in cancer-associated fibroblasts in human lung cancer tissues and spontaneous lung tumors in mice. Knocking out or inhibiting HIF-1α significantly attenuated fibroblast activation by downregulating NF-ĸB signaling with significant reduction of CCL5, a potent pro-inflammatory chemokine and restricted tumor growth [25].

**Figure 1:**
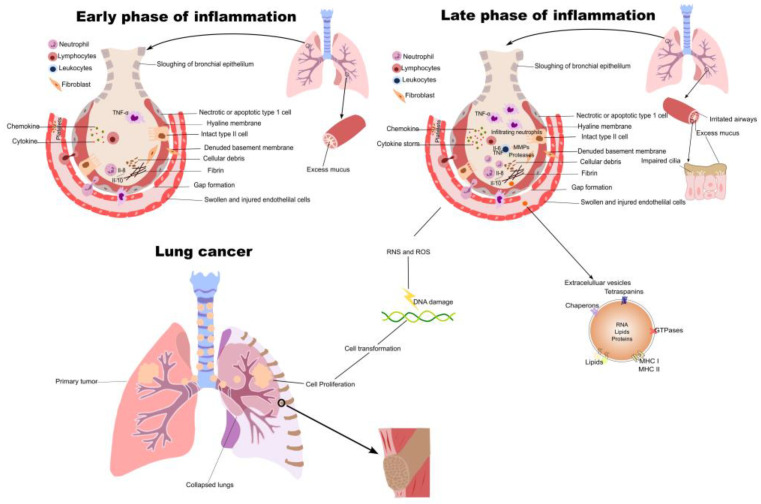
Neutrophils have a significant impact on early event of acute lung inflammation. The formation of granulation tissue, which is made up of cellular matrix, fibroblasts, endothelial cells, and leukocytes, is orchestrated by neutrophil migration and chemokine production during acute inflammation. Inflammation becomes chronic when acute inflammation is not treated. Other inflammatory immune cells, such as macrophages and lymphocytes, intensify lung inflammation during chronic inflammation, increasing the risk of cancer by fostering tumorigenesis at all stages, including initiation, invasion, and metastasis. Lung cancer progression is also aided by inflammation because it supplies the tumor microenvironment with vital molecules by the help of extracellular vesicles.

Acute lung inflammation is an early event where neutrophils play a major role. During acute inflammation, neutrophils migration and chemokine production initiate granulation tissue formation comprising endothelial cells. cellular matrix, fibroblasts, and leukocytes [12]. When the acute inflammation is not resolved, chronic inflammation takes place. During chronic inflammation, other inflammatory immune cells such as macrophages and lymphocytes induce a more severe inflammation in lung, which eventually elevates cancer risks by promoting all stages of tumorigenesis (Figure 1). Tumors arising from chronic inflammation regions are often described with infiltrating leukocytes (mostly macrophages), growth factors, cytokines, and metastatic-promoting enzymes. Depending on type of immune cell infiltrate, the infiltrating leukocytes can exert either pro-tumor or antitumor effects.

A hallmark of lung inflammation is immune cell infiltration. Immune cells infiltrate can be divided into innate and adaptive immune cells [26]. Innate immune cells, such as dendritic cells, myeloid-derived suppressor cells (MDSCs), TAMs, and neutrophils, can either promote or suppress tumor initiation and progression during tissue injury [27]. Dendritic cells mediate antitumoral immunity by cross-presenting tumor antigens to activate T-lymphocytes in lymph nodes [28]. MDSCs degrade L-arganine, and inhibit NK cell-derived IFN-γ production as well as CD4 and CD8 T cell IFN-γ production [29]. MDSCs curb T cell activity by downregulating pro-inflammatory cytokines, such as prostaglandin E2 (PGE2) and IL-12, reducing host immunity to target cancerous cells (Huang et al., 2019). Anti-CD33 antibodies such as Gemtuzumab and ozogamicin were able to restore T cell immunity against cancer [30]. Accumulation of MDSCs is boosted by HIF-1α, released within the hypoxic condition of TME, to facilitate immune evasion of tumor cells [31]. TAMs can be divided into macrophage 1 (M1) and macrophage 2 (M2). M2 macrophages are usually associated with tumor progression, whereas M1 macrophages generate reactive oxygen and nitrogen intermediates, which induces DNA damage in proliferating cells leading to neoplastic transformation. The biological behavior of A549 NSCLC cells, after co-culturing with various macrophage subtypes, were examined in one such study. In this study, M2 macrophages stimulated A549 cell invasion and tumor growth and in contrast, M1 macrophages inhibited A549 cell proliferation and viability by triggering apoptosis and senescence. The results from this study suggested an increased expression of DNA damage-induced proteins, where GADD34 and GADD153 in M1-A549 cells are highly susceptible to cisplatin [32].

Under normal physiology, inflammatory cytokines are released by macrophages to stimulate repair of damaged cells [33]. However, overexpression of inflammatory cytokines, chemokines, and growth factors by TAMs inhibited key enzymes, such as xanthine oxidase (XO), NADPH oxidase, and NOS, which induce accumulation of DNA-damaging agents and disrupt genome integrity and stability [14,33]. TNF-α, IL-6, IL-1β, and IL-8 are often secreted by macrophages in response to smoking or inhaling toxic chemicals from cigarette smoke [34]. Accumulation of TNF-α triggers angiogenesis cascade by inducing “tip-cell” phenotype in endothelial cells with the help of an NF-κB-dependent mechanism [35]. IL-6 plays the role as an activator of JAK and STAT 3 [36] as well as inducing lung cancer metastasis [37]. In chronic inflammation, neutrophils promote carcinogenesis either by supporting tumor-related inflammation, angiogenesis, and metastasis or restricting tumor growth through expressions of antitumor and cytotoxic mediators [38]. Accumulation of neutrophils in the lung enhances lung metastasis by boosting release and production of MMP9. The surge increase of MMP9 by neutrophils results in breakdown of collagen and induce production of inflammation-generated extracellular matrix fragments ac-PGP (N-acetyl-proline-glycine-proline), which act as chemoattractant to stimulate cancer cell migration [39]. Other from MMP9, degranulation of azurophilic granules in neutrophils also elevates other enzymes, such as Ser proteases, cathepsin G, and elastase, leading to degradation of antitumorigenic factor thrombospondin-1 (Tsp-1). Without Tsp-1, tumor growth in metastatic organs will be uncontrollable [40]. Furthermore, neutrophils also assist in contributing neo-plasticity in TME by releasing reactive oxygen species (ROS), thus favoring tumor formation [12].

Apart from changes in innate immunity, adaptive immune cells such as T-lymphocytes, immunosuppressive FOXP3+ T regulatory cells, and CD8+ cytotoxic T-cells are involved in lung cancer development and progression [26]. T-lymphocytes execute cytotoxic effects in tumor microenvironment, and these responses are linked to immune checkpoint inhibition (ICI). Immunosuppressive FOXP3+ T regulatory cells, induced by cyclooxygenase 2 (COX-2), can enhance tumor burden [41]. FOXP3 is essential for Treg development and differentiation. Tregs coordinate cellular and molecular networks to create an immunosuppressive environment and encourages tumorigenesis [42]. CD8+ cytotoxic T cells secrete cytotoxic molecules or make cell-to-cell contact to induce tumor cell apoptosis [43]. Gamma-delta (γδ) T cells are a type of T cells that initiate inflammatory responses of myeloid and lymphoid cell lineages. These γδ T cells can be easily activated by inflammation provoked by local microbiota, leading to the development of lung adenocarcinoma. Germ-free KRAS-mutated and p53 null mice were found protected from lung tumor development after treatment with combination of antibiotics (ampicillin, neomycin, metronidazole, and vancomycin). It was postulated that local microbiota stimulated production of IL-23 and IL-1β in myeloid cells were responsible for the proliferation and stimulation of γδ T cells in these mice. Activated γδ T cells also produce and release IL-17 and other effector molecules to induce inflammation and tumor proliferation [44].

Inflammation supports lung cancer progression by providing essential molecules to the TME. This is achieved with the help of extracellular vesicles (EVs). EVs are lipid bilayers released by a variety of cells. They are also called macrovesicles, exosomes, or apoptotic bodies [45]. EVs harbor cargo molecules, such as RNA, lipids, and proteins, and these cargo molecules are transferred to recipient cells, acting as intercellular communicators within lung TME. Smoking is a major risk factor for lung cancer. In a recent study, smoking-induced extracellular vesicles were characterized in NSCLC smokers, where higher amounts of EVs were found in bronchoalveolar lavage. Furthermore, long non-coding RNAs (MALAT1, FOXD2-AS1, HOTAIR, HOTTIP, HOXA11-AS, AGAP2-AS1, ATB, TCF7, PCAF1, and BCAR4) were detected in smoke-induced EVs and were significantly higher in NSCLC smokers versus non-smokers. In the same study, signaling pathways involving proteoglycans, ErbB, fatty acid biosynthesis, Hippo, Rap1, TGF-β, Wnt, AMPK, and Ras were heavily enriched in EVs from NSCLC patients through bioinformatic analysis identification [46]. The oncogenic contents in EVs could further drive disease progression, as they could be easily absorbed by other cells and influence cellular programming at transcriptional and post-transcriptional levels. These EVs can further inflict damage by attracting cancer-associated fibroblasts, promoting angiogenesis and remodeling of the extracellular matrix to support metastasis within TME [45]. Release of these cargo molecules within the TME changes inflammatory cytokines and growth factor levels, such as transforming growth factor (TGF)-β, IL-1β, IL-6, IL-4, IL-11, IL-12, and MCP-1, and TNF-α activating pro-inflammatory signaling cascades, such as MAP kinases and NF-ĸB pathways [47]. EVs produced by vascular and blood cells can also contribute to the development of atherosclerosis in several ways. Together with LDL cholesterol, they increase thrombotic risks by promoting inflammation, vascular dysfunction, leukocyte adhesion, and tissue remodeling [45].

## 3. Inflammatory Cytokines in NSCLC

Both TGF-β and IL-6 are produced in response to inflammation in NSCLC. In erlotinib-naïve NSCLC-derived cell lines and early-stage NSCLC tumors, intrinsic erlotinib-resistant cell subpopulations displayed features suggestive of epithelial-to-mesenchymal transition (EMT). TGF-β is a potent driver of EMT in most cancers. Activation of TGF-β signaling induces not only the EMT phenotype, but also promotes TGF-β-dependent IL-6 secretion, which elevates inflammation in TME. Increased inflammatory response in TME has been shown to adversely affect tumor response to EGFR-TKI, erlotinib [48]. Eph receptors participate in tumor progression and their expression is governed by the TGF-β-activated Smad 2 pathway. Eph was found upregulated in NSCLC patient biopsies and significant increase of Eph receptor expression was found correlated with poor survival [49]. Nevertheless, the role of EpH in NSCLC via inflammation is yet to be fully explored. IL-6 is a multipotent pro-inflammatory cytokine, which activates the JAK-STAT3 signaling pathway. In a cancer context, it plays a significant role in multiple tumors, such as lung, colon, breast, prostate, ovarian, and multiple myeloma [36]. Fibroblasts, isolated from human lung cancer tissues, were found to actively secrete IL-6 and enhance metastatic activity in human lung cancer cell lines through JAK2 and STAT3 signal transduction activation [37]. In K-ras mutant lung cancer mouse model, IL-6 was found overexpressed and blocking IL-6 with monoclonal antibody significantly reduced airway inflammation and dampened lung tumor cell proliferation and angiogenesis [50]. However, another study highlighted that IL-6 deficient mice developed higher numbers but smaller-sized lung tumors after activation of mutant KRAS in lung. IL-6 could prevent growth of lung tumors in the early cancer stage by maintaining lung homeostasis through regulation of lung macrophages and cytotoxic CD-8 T cells with IL-6/STAT3 signaling activation promoted tumor progression with more tumor colonies through induction of cell proliferation regulator cyclin D1 under KRAS oncogenic stress [51]. As such, it is vital to keep optimal IL-6 levels to avoid disease progression.

IL-1β, mainly produced by myeloid cells, such as macrophages, mast cells, and neutrophils, regulate various cellular activities including cell differentiation, differentiation, apoptosis, and proliferation. This pro-inflammatory cytokine is often found in lungs of patients with COPD and asthma [52]. The CANTOS trial (Canakinumab Anti-inflammatory Thrombosis Outcomes Study) was a clinical trial conducted to evaluate the safety and efficacy of canakinumab, a monoclonal antibody which targets IL-1β. This trial was conducted in patients with a history of myocardial infarction and high levels of high-sensitivity C-reactive protein (hsCRP), an inflammatory biomarker. Besides reducing the recurrence of cardiovascular events compared to placebo group, the results also indicated that canakinumab significant impact on lung cancer. CANTOS trial revealed IL-1β and inflammasome inhibition could significantly lower incidents of lung cancer [53]. This notorious inflammatory cytokine also promotes blood vessels and lymphatic angiogenesis after inflammasome activation to support tumor development [54]. Angiogenesis is required for tumor progression through formation of new blood vessels to facilitate long distance migration of tumor cells. Pro-inflammatory cytokines including TNF-α and IL-1β activate NF-κB pathway, a key regulator of cell proliferation and growth. However, in tumor cells, persistent NF-κB activity in TME induces angiogenesis and apoptosis, and promotes tumor cell invasion and EMT. Furthermore, it can elevate cyclin D and E expressions, which increases the transition from G1 phase to S phase of dividing cells [14].

IL-8 signaling axis is associated with pathogenesis of inflammatory-based diseases, including cystic fibrosis, asthma, chronic obstructive pulmonary diseases (COPD), and cancer. Secretion of IL-8, by tumor and other cells within the stroma, is critical for cancer progression and metastasis [55]. In NSCLC, epigenetic modification of IL-1β, IL-8, and IL-6 expressions could affect inflammatory response during cancer development [56]. The same observation was seen in tumor cells from NSCLC patients, where rapid lowering of IL-8 serum levels were observed after surgical tumor excision. In human NSCLC, serum IL-8 levels were found correlated with tumor burden and could be utilized as a biomarker to predict tumor burden [57].

TNF-α is a notorious inflammatory cytokine and is often associated with hormone non-responsiveness, poor prognosis, and cachexia [58]. It plays an important role in activating the NF-κB signaling pathway in tumor promotion [59]. TNF-α promotes pleural effusion of lung cancers by causing excessive permeability of airway blood vessels [60]. Surprisingly, TNF-α has been proposed to be used for cancer treatment, as it possesses the ability to induce vascular hyperpermeability and destruction of vascular lining in tumor-associated vasculature. This strategy should aid the accumulation of administered cytotoxic drugs in tumor after vasculature destruction [61].

MCP-1 regulates monocyte chemotaxis and lymphocyte differentiation through CC chemokine receptor 2 (CCR2) binding and plays a significant role in pathogenesis of inflammatory diseases, such as asthma, COPD, and cancer [62]. In the cancer microenvironment, cancer cells and non-cancerous stromal cells, including inflammatory cells, endothelial cells, and fibroblasts, produce MCP-1, which enhances cancer cell migration, survival, and proliferation [63]. MCP-1 expression in solid tumors were evaluated through meta-analysis, and results showed that high level of MCP-1 were related with decreased survival rate (hazard ratio 1.95, 95% CI 1.32–2.88) [64]. In bone cancer, MCP-1 enhances metastasis by promoting interaction between host-derived chemokines and tumor-derived factors [65]. Table 1 shows a summary list of common targets of inflammation in NSCLC.

## 4. Drugs and Molecules Targeting Inflammation in NSCLC

Inflammation occurs in both early and late phases of lung cancer, and is a highly complex process. The proteins involved in this process can be utilized as candidate targets to treat lung cancer. Targeting inflammation can be applied both to lung cancer induced by intrinsic factors and extrinsic factors.

Mutations in leucine-rich-repeat kinase 2 (LRRK2) are common in immune-related disorders, such as inflammatory bowel disease and Parkinson’s disease [77]. LRRK2 can modulate inflammation during microbial infection in mouse model. LRRK2 mutations are associated with worsened survival of infected animals [78]. LRRK2 is highly expressed in immune cells and has important roles, including regulation of cytokine release, autophagy, and phagocytosis [77]. In a recent study, loss of LRRK2 was observed to promote carcinogen-induced lung tumorigenesis in both patient and mouse lung cancer models. In NSCLC patients, reduced LRRK2 levels led to immunosuppression, altered surfactant metabolism, and lessened differentiated lung adenocarcinoma. It is proposed that the developmental program of growth and differentiation of tumor is strongly associated with weakened activation of inflammatory activities within the region. This observation was fully supported in a carcinogen-induced murine lung cancer model, where LRRK2 knockout led to a significant increase of both tumor numbers and sizes [75]. LRRK2 kinase and GRPase inhibitors, such as MLi-2, PF-06447475, GNE-0877, compound 68 and 70, and FX2149, initially developed for treatment of Parkinson’ disease [79], could be repurposed to treat LRRK2-associated lung cancer.

Isochorismatase domain containing 1 (ISOC1) is a potential biomarker in gastrointestinal cancer, but its role in cancer remains unknown [73]. ISOC1 has also been reported to regulate the growth of breast and pancreatic cancer cell lines. ISOC1 knockdown in these cell lines reduces growth and cell proliferation, induces cell apoptosis, and elevates caspase-3/7 [80]. Elevated ISOC1 expression was seen in NSCLC patients with records of unfavorable disease-free survival. ISOC-1 overexpression in NSCLC cells induced cell proliferation, viability, migration, and invasion, whereas ISOC1 knockout in mouse xenograft model led to significant tumor growth inhibition [73]. ISOC1 suppression also inhibited cell proliferation and migration and induced apoptosis in colon cancer cells [81]. Using RNA sequencing analysis, signaling pathways mediated by ISOC-1 were mainly inflammation related [73].

The enzymatic subunit of polycomb repressive complex 2 (PRC2) is known as an enhancer of zeste homolog 2 (EZH2) and has been identified to activate oncogenes, inhibits tumor suppressor factors, promoting metastasis, altering immunity and metabolism, as well as inducing drug resistance [82]. NSCLC tumors were found to possess high levels of EZH2. In orthotropic KRAS-driven EZH positive NSCLC grafts, treatment with EZH2 inhibitor GSK126 could amplify inflammation through activation of NF-ĸB and genes residing within the PRC-2 regulated chromatin. The inflammation allowed tumor cells to overcome GSK126 antiproliferative effects, an unfavorable event and possibly rendering EZH2 inhibitors ineffective against KRAS-driven NSCLC. In the same study by Serresi et al., GSK126-treated NSCLC in vivo displayed enhanced response towards nemisulide (NSAID) and bortezomib combination treatment [74]. Aspirin, naproxen, sulindac acid, amino salicylic acid, and celecoxib are NSAIDs that should be considered for use in combination with EZH2 inhibitors for KRAS-driven NSCLC. These lines of evidence indicate that combination of anti-inflammatory drugs is a plausible strategy to resolve EZH inhibitor ineffectiveness in KRAS-driven NSCLC.

Molecules targeting signaling pathways such as NF-ĸB and STAT3 are often studied in inflammatory-based diseases including cancer. STAT3 pathway transmits extracellular signals to the nucleus and regulates immunity, inflammation, and tumorigenesis. Activation of STAT3 mediates various cellular processes including survival, proliferation, invasion, inflammation, angiogenesism and metastasis [83,84]. STAT3 pathway activation in NSCLC induces tumor resistance towards conventional and small molecule targeted therapy [85]. STAT3 often interacts with other signaling pathways, such as NF-ĸB, commonly associated with lung inflammation and confers robustness in tumor progression. Hyperactivation of STAT3 leads to a series of tumor promoting events, such as immunosuppression in tumor-infiltrating cells, dampening antigen presentation, and inhibition of tumor-killing activities [86]. Existing drugs targeting the NF-ĸB pathway, such as thioridazine, imatinib, mesylate, clemastine, and ibudilast, can be repurposed for lung cancer treatment [87,88,89]. Imatinib, an oral anticancer agent that inhibits tyrosine kinase activity, is used to inhibit BCR-ABL1 fusion oncoprotein, c-kit, platelet-derived growth factor receptor (PDGFR), and native tyrosine-protein kinase Abelson murine leukemia (ABL1 kinase) [90]. Imatinib can modulate immune response by inhibiting IL-6 and other proinflammatory cytokines through suppressing NF-ĸB activity [90]. Two clinical phase 2 trials of Imatinib, in combination with docetaxel or paclitaxel, reported poor clinical outcomes due to poor therapeutic responses and unwanted side effects, such as chronic gastrointestinal toxicity (nausea, vomiting, and diarrhea) and cardiac toxicity (cardiomyocyte injury) [91,92]. The antagonistic effects of Imatinib suggest that caution should be taken administering in combination with other drugs. However, the unwanted side effects can be reduced if Imatinib is given directly to the lung instead of the usual i.v. route.

Pro-inflammatory cytokines are important in ensuring that inflammation is regulated at an optimal level to promote lung carcinogenesis. Lowering pro-inflammatory cytokines, such as IL-1β, IL-2, IL-6, IL-8, IL-10, IL-12, interferon γ (IFN-γ), TNF-α, and granulocyte-macrophage colony-stimulating factor (CSF), can reduce lung cancer risk, particularly among smokers [93]. These pro-inflammatory cytokines can be inhibited with existing biologics such as antibodies targeting either a cytokine or its receptor. Sarilumab, Tocilizumab, and siltuximab are existing FDA-approved IL-6 inhibitors for rheumatoid arthritis and COVID-19 to reduce damages caused by IL-6-induced inflammation [94,95]. On the other hand, glucocorticoids, which are wide-spectrum anti-inflammatory agents, reduced pro-inflammatory cytokine expression via genomic and non-genomic pathways in COVID-19-induced acute respiratory distress syndrome (ARDS) patients [96]. In addition to that, dimethyl fumarate (DMF) also inhibits more extensive pro-inflammatory cytokines, especially IL-1 and IL-6 [97]. Three IL-1 inhibitors (anakinra, rilonacept, and canakinumab), used as a single agent or in combination for treatment of rheumatoid arthritis and IL-1, induced autoimmune disease [98], and can be utilized to reduce IL-1 induced lung inflammation. Results from a recent CANOPY-1 Phase III study showed that locally advanced or metastatic NSCLC patients treated with canakinumab did not achieve its primary endpoints of overall survival and progression-free survival. However, the study recommended the use of canakinumab in patients with elevated inflammatory biomarkers at early stages of lung cancer, as canakinumab-treated patients showed improved progression-free survival and overall survival [99].

PD-1 immune checkpoint pathway is an attractive NSCLC therapy where it prevents T-cell activation by downregulating immune system response, promoting self-tolerance and reducing auto-immunity. However, the PD-1/PD-L1 pathway has also been associated with significant inflammatory effects. Besides cancer, this pathway has served as a target in other inflammatory-based diseases, including autoimmune responses, chronic infections, and sepsis [100]. Nivolumab, a human immunoglobulin G4, has demonstrated superior overall survival in patients with advanced squamous NSCLC patients when compared to docetaxel [3]. In cancer immunotherapy, many inhibitors of pro-inflammatory cytokines, such as TNF-α, TGF-β, and CSF, have been used in combination with anti-PD-L1 or anti-PD-1 agents, and have shown promising improvement in therapeutic outcomes, in comparison to monotherapy agents Cytokines, which exert therapeutic efficacy by potentiating immune response and inhibiting the immunosuppressive activity. Currently, there are ongoing clinical trials to evaluate therapeutic effect of cytokines by combining them with various anti-PD-L1 and anti-PD-1 agents [101].

Neutralizing the effects of pro-inflammatory cytokines with existing biologics, small molecules, cytokine traps, or RNA interference should be further explored. Interestingly, expressions of estrogen receptor, progesterone receptors, and aromatase have been associated with poor prognostic outcome in post-menopausal NSCLC in females [102]. In a tobacco carcinogen-induced lung tumor mouse model, the combination of aromatase inhibitor (anastrozole) with an NSAID (ibuprofen or aspirin) resulted in stronger tumor prevention effects in comparison to a single agent. The combination treatment reduced infiltrating macrophages, deactivated MAPK and STAT3 signaling, and inhibited inflammatory markers, such as IL-6 and IL-17A [102].

Excessive production of ROS can damage normal and cancer cells and regulate the production of oxidants at an optimal level for survival. The ROS produced activate many oncogenes, which induces production of inflammatory cytokines and factors. Statins are low-density lipoprotein cholesterol lowering drugs that inhibit enzyme HMG-CoA reductase. Rosuvastatin was reported to inhibit pro-inflammatory cytokines including tumor necrosis factor-α (TNF-α), IL6, and TGF-β in mice, leading to tumor shrinkage [103]. Liposomal pravastatin treatment was found to effectively inhibit inflammatory cytokine production, such as GM-CSF, IGF-II, IL-1α, IL-1β, leptin, IL-6, and TNF-α [104]. A recent study investigated the association between statin exposure and lung cancer risk in a population of COPD patients (n = 39,879) and the results showed that statin use significantly reduced the risk of lung cancer The results indicated that statin use may specifically reduce non-small cell lung cancer and not small cell lung cancer. Lung cancer risk is reduced by statins via a reduction in systemic inflammation, which also leads to slowing down of the decline in lung function and reduction in all-cause mortality [105]. Despite possessing potent anti-inflammatory effects, the exact anti-inflammatory mechanism of statins is unknown. Statins are known to exert pleiotropic effects, such as reducing cell proliferation, angiogenesis, and invasion. However, given statin’s potency, this drug remains highly attractive for the prevention and treatment of lung cancer [103].

The hypoxia-inducible factor (HIF) pathway plays a crucial role in solid tumors including lung cancer. Among the two sub-units HIF-α and HIF-β, HIF-α plays a more crucial role in regulating inflammation [23,24,25]. There are several suitable HIF inhibitors currently in phase II and III of clinical trials, and results have shown promising outcomes for gliobastoma, melanoma, lymphoma, colorectal, and mesothelioma malignancies. HIF-α inhibitors, including 2ME2 NCD (panzem), 17-AAG (tanespimycin), Vorinostat (SAHA), EZN-2208 (pegylated SN-38), and CRLX101, are potential drugs to be considered for the treatment of lung cancer [31].

**Figure 2:**
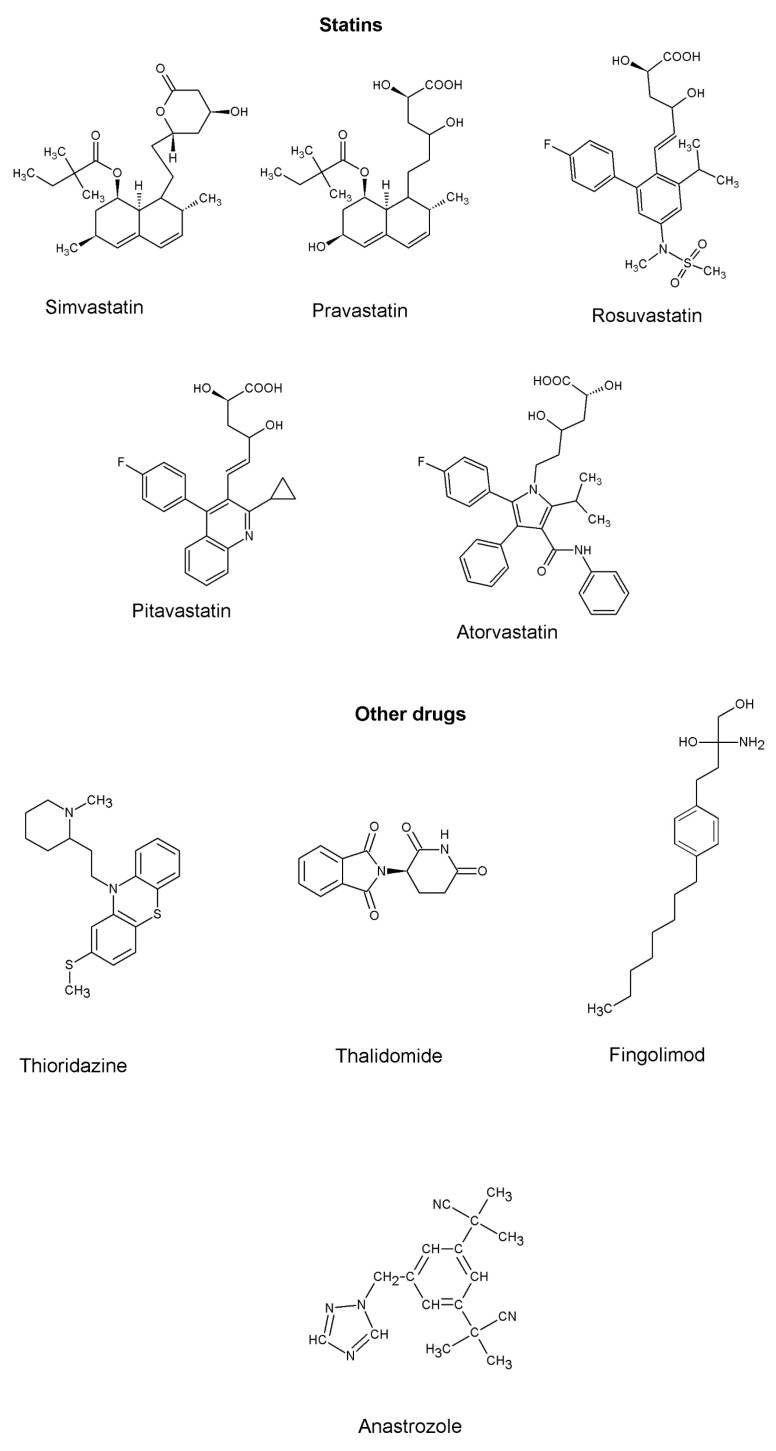

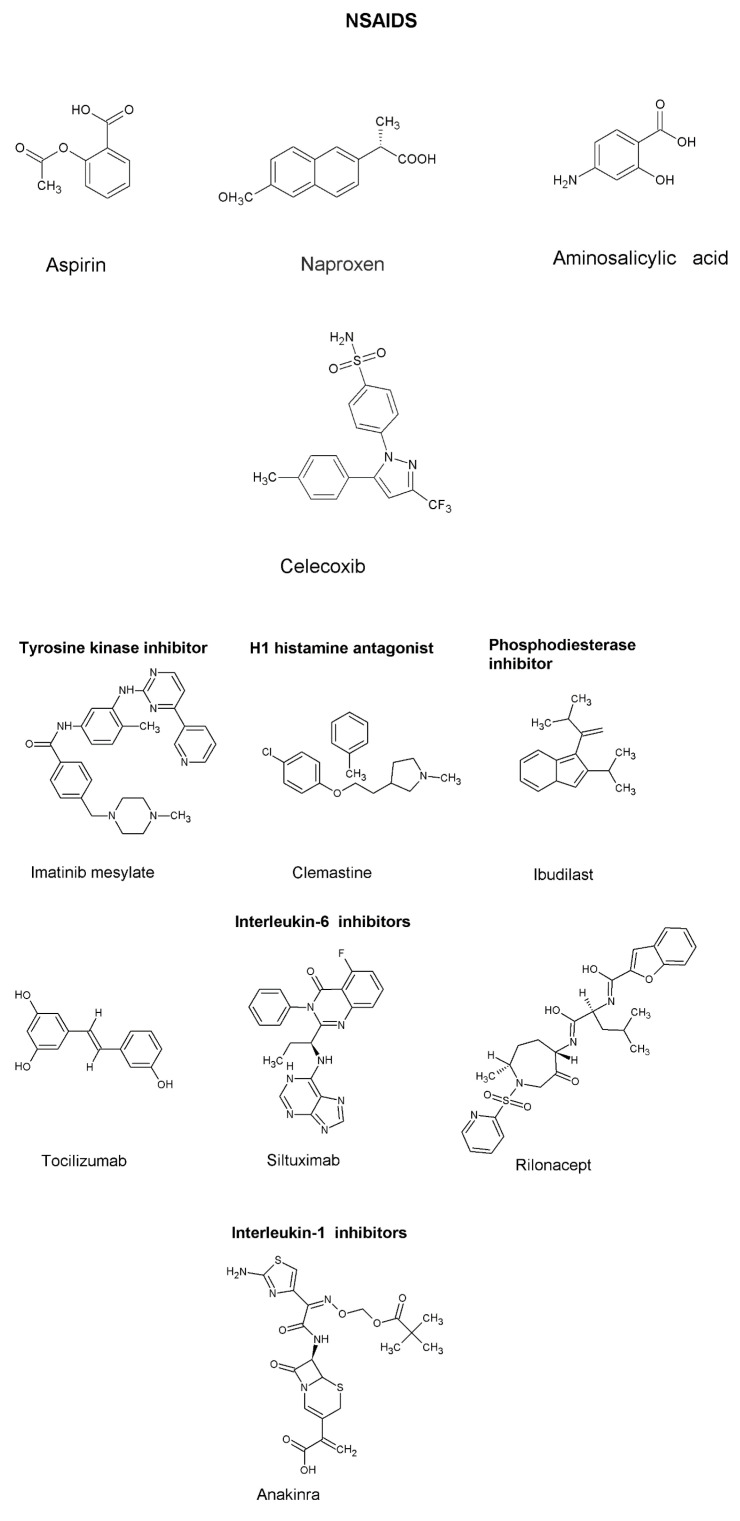

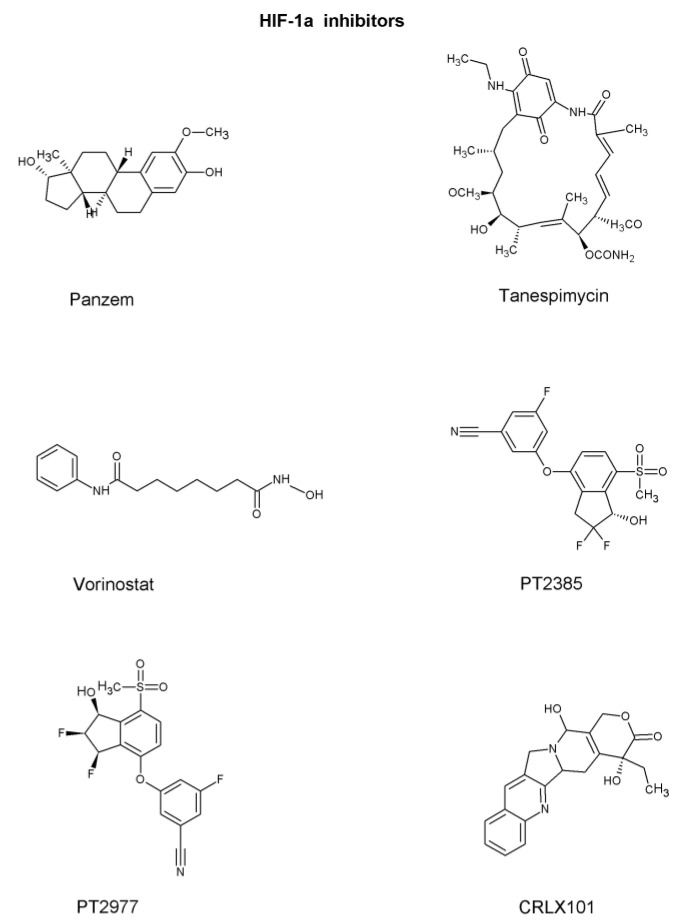
Chemical structures of the drug to be repurposed.

**Figure 3:**
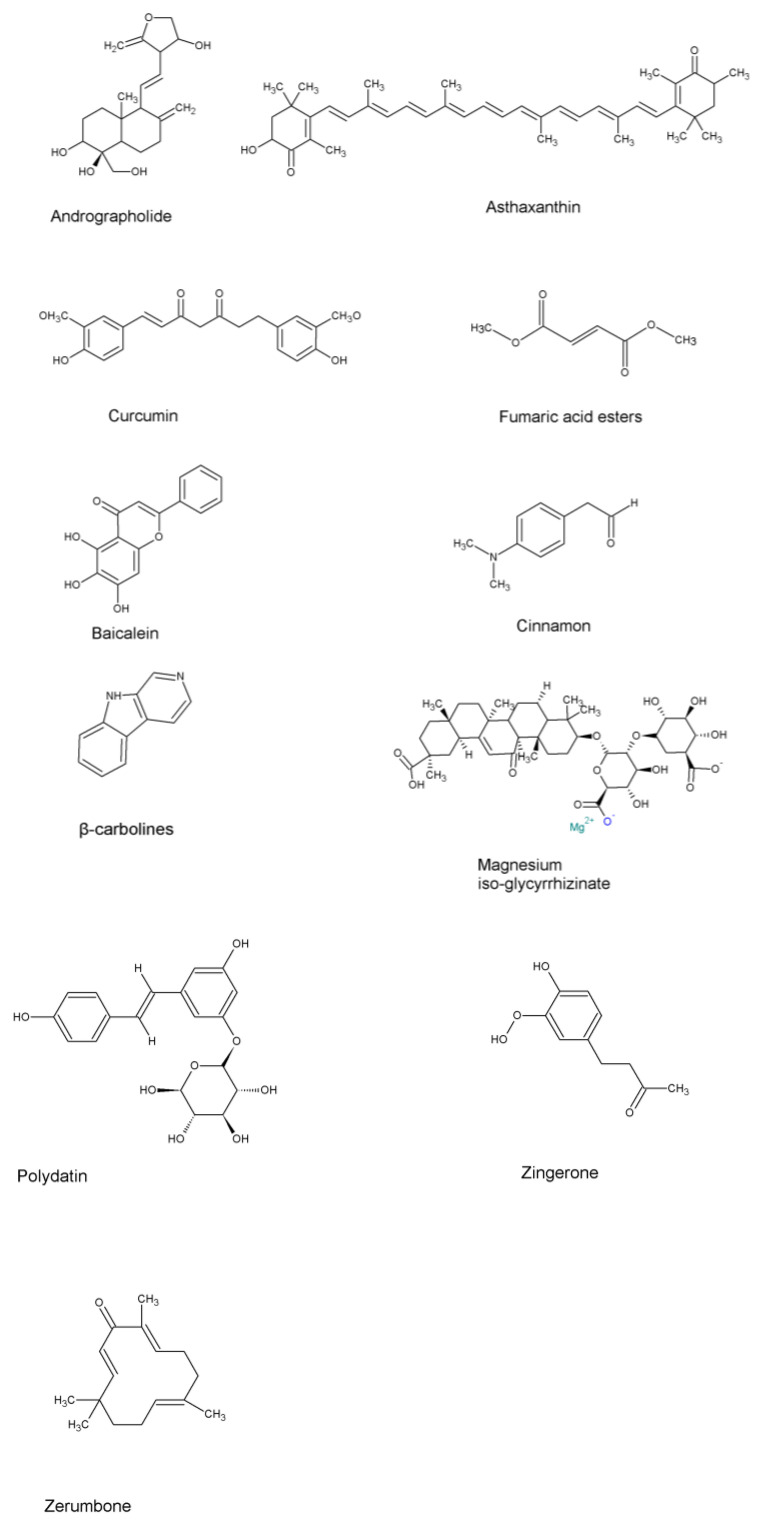
Chemical structures of the natural compounds to be repurposed.

Fingolimod (FTY720), a sphingosine-1-phosphate receptor modulator, is commonly used as an immunomodulator in multiple sclerosis treatment [106]. Inflammatory mediators, such as TNF-α, IL-6, and IL-1β, were found induced in rats with renal ischemia/reperfusion, which resulted in lung injury. Intraperitoneal FTY720 administration were found to protect against acute lung injury by reducing pulmonary inflammation through downregulation of sphingosine-1-phosphate metabolism [73]. Additionally, FTY720 also prevented pulmonary cell apoptosis in a renal ischemia/reperfusion model [73] and induced fibrosis in a bleomycin-induced lung injury model [107], indicating that a combination of strong apoptosis inducer and antifibrotic agents could be given for effective lung cancer treatment. Table 2 provides a summary of existing drugs with anti-inflammation properties for NSCLC treatment and their chemical structures are included in Figure 2. Table 3 shows natural compounds that are currently undergoing clinical trials for the treatment of non-small cell lung cancer and their chemical structures are included in Figure 3.

## 5. Natural Compounds Targeting Inflammation in NSCLC

Natural products remain a potential source for new and innovative drug discovery, given that many have shown to possess anti-inflammatory properties. They are rich with secondary metabolites, such as flavonoids, terpenes, and alkaloids. Several herbal medicinal plants have been actively studied for their anti-inflammatory properties. In recent years, many natural products have been reported to exert effects against lung TME. Most are recognized with potential to be developed as new plant-derived chemotherapy agents due to their ability to modulate angiogenesis, the extracellular matrix, MDSC, TAMs, and immune checkpoint [113].

Cinnamon contains secondary metabolites, such as cinnamaldehyde, cinnamic acid, 2-hydroxycinnamaldehyde, 2-methoxycinnamaldehyde, and eugenol, and possesses potent anti-inflammatory effect by reducing pro-inflammatory IL6, IL-1β, and TNF-α and suppressing NF-ĸB-mediated COX-2 and iNOS pathways [114]. With regards to NSCLC, cinnamon extract was found to suppress invasion of A549 and H1299 cells by regulating the expression of FAK and ERK pathways [115]. Combination therapy with cinnamaldehyde and hyperthermia was also found to induce apoptosis of A549 cells by regulation of reactive oxygen species and the MAPK pathway [116].

Hochuekkito (TJ-41) is a Japanese traditional herbal kampo medicine comprising 10 natural herbs. TJ-41 was shown effective in attenuating lung inflammation in the COPD mouse model and LPS-induced macrophage cell line through TNF-α ablation [117]. Secondary plant metabolites found to possess potent anti-inflammatory properties include Andrographolide, baicalein, curcumin, Pterostilbene, Dihydroisotanshinone I, Ginsenoside Rh2, vitamin D, and zerumbone.

Immune cell regulation is a crucial event in lung inflammation, and manipulating these immune cells can prevent inflammation and impede cancer progression, particularly in the early stages. Andrographolide, the primary active component found in Andrographis paniculata, is a labdane diterpene known for its potent anti-inflammatory properties [118]. It has been reported to inhibit the production of several pro-inflammatory cytokines and chemokines, such as TNF-α, IL-6, and IL-8, and suppress the activation of the NF-κB and MAPK signaling pathways, which are crucial regulators of inflammation [119,120]. Additionally, andrographolide has been found to exhibit anticancer effects in various cancers.

Andrographolide has been reported to suppress migration of macrophages towards chemo-attractants, such as complement 5a (C5a), through the inhibition of phosphorylation of mitogen-activated protein kinase (MAPK) kinase 1/2 (MEK1/2) and downstream p42/p44 MAPK (aka extracellular signal-related kinase 1/2, ERK1/2) and Akt signaling pathways [121]. Furthermore, andrographolide acts on other cellular pathways regulation, including mTOR, Wnt/β-catenin, TRAIL-mediated apoptosis, as well as VEGF-mediated intracellular signaling, and adversely affects tumor development [122]. Interestingly, andrographolide can also inhibit human NSCLC cellular proliferation and induces apoptosis by reprogramming host glucose metabolism [123]. In addition to growth inhibition, andrographolide could suppress aggressive metastatic cancer, including luminal-like breast cancer through NF-ĸB pathway inhibition [124]. Data from high-throughput metabolomics analysis revealed that this compound exerted its anticancer properties by enhancing immune system activity, reduces inflammation, tumor cell metastasis, and balancing visceral metabolism in a Lewis lung cancer model [125]. Resistance to cisplatin in NSCLC, achieved through autophagy, is a hindrance and andrographolide is found capable of inhibiting autophagy in cisplatin-resistant NSCLC by activating the Akt/mTOR pathway, and re-sensitizes tumor cells towards cisplatin [126].

Pterostilbene, Dihydroisotanshinone I, and Ginsenoside Rh2 are natural compounds capable of inhibiting TAMs activity [113]. Pterostilbene is a natural analogue of resveratrol, which has metabolic stability and superior pharmacological activities. Pterostilbene was initially extracted from red sandalwood (Pterocarpus santalinus) and can primarily be found in a few natural sources, such as grapes, blueberries, and *Pterocarpus marsupium* [127]. Pterostilbene treatment led to reduced expressions of NF-ĸB, CD133, MUC1, β-catenin, and Sox2 in inflammatory lung TAMs. This also led to a significant loss of stemness by TAMs with decreased side-population cells and suppression of self-renewal ability in TAM-co-cultured lung cancer cells [128]. In another study, female Balb/C mice were treated with varying doses of pterostilbene to examine its impact on cell proliferation, cell death, and the p53 pathway. The study observed a reduction in Ki-67 expression and an increase in caspase-3 expression, leading to a decrease in cyclin D1 and cyclin E2 protein expression, causing cell cycle arrest. Furthermore, pterostilbene increased p53, p21, and p27 protein expression [127].

Dihydroisotanshinone I is a pure compound extracted from danshen, which is a Chinese medicinal herb. Dihydroisotanshinone I could inhibit tumor migration and cell motility, block macrophage recruitment by lung cancer cells, reduce CCL2 secretion, and suppress p-STAT3 signaling in NSCLC A549 and H460 cells [129].

Ginsenoside Rh2, found in ginseng, possessed the ability to convert TAMs from M2 to M1 subtype and prevented cancer cell migration by curbing TAMs activity in TME [130]. Ginsenoside Rh2 was also found to inhibit hypoxia-induced cell migration by increasing the expression of mir-491, which subsequently downregulated the expression of MMP-9. In this study, the effects of ginsenoside Rh2 on hypoxia-induced migration in lung adenocarcinoma was studied. Rh2 was found to inhibit hypoxia-induced cell migration in A549 and H1299 cell lines through the upregulation of mir-491 expression. Additionally, mir-491 antisense oligonucleotide suppressed hypoxia-induced migration and the expression of matrix metalloproteinase (MMP)-9 expression in Rh2-treated A549 cells [131].

Vitamin D, found in sea fish and animal liver, was reported to reduce hyperinflammation in both macrophages and MDSCs in COVID-19 patients. A complication, commonly seen in the lungs of COVID-19 patients, is hyperinflammation-induced acute respiratory distress syndrome. In patients who suffered from acute respiratory distress syndrome and lacked vitamin D, symptoms were reduced after vitamin D supplementation [132]. Vitamin D was also found to improve the survival of patients with cancer found by a meta-analysis of randomized clinical trials performed [133]. Additionally, in a randomized controlled trial with 155 patients NSCLC, vitamin D supplementation show a significant difference in relapse-free survival (RFS) or overall survival (OS) compared to placebo among the subgroup of patients with early-stage adenocarcinoma and low levels of 25(OH)D [134].

Resveratrol is a naturally occurring non-flavone polyphenol compound that is derived from various plants, such as *Polygonum cuspidatum*, *Cassia tora Linn*, and *Vitis vinifera*. It belongs to the stilbene family. Resveratol was found to activate autophagy and apoptosis in A549 cell by regulating the NGFR-AMPK-mTOR signaling pathway [135]. Resveratrol, found in grape skin and seeds, and silymarin, from Silybum marianum, were shown to modulate MDSCs in lung cancer in vivo model [113].

Baicalein is a major bioactive compound found in the root of *Scutellaria baicalensis*, a traditional Chinese herb. Baicalein was reported to effectively inhibit NSCLC cell invasion and metastasis without any toxicity. This flavonoid significantly reduces ezrin tension by reducing cellular ezrin S-nitrosylation (SNO) levels and iNOS expression in the inflammatory microenvironment of NSCLC [136]. Baicalein has been proven to exert anti-airway inflammation in cigarette smoke-induced chronic obstructive pulmonary rat model by regulating pro- and anti-inflammatory balance [137], and in an OVA-induced allergic airway inflammation model through iNOS and NF-ĸB signaling inhibition [138]. Dimethyl fumarate is a promising fumaric acid ester and possesses strong anti-oxidative, anti-inflammatory, and immunomodulation properties [139]. Given to mice with chronic exposure to diesel exhaust particles, peroxynitrite, total reactive oxygen species, and nitric oxide levels in the lung were significantly reduced, whereas expression of products such as nitrotyrosine, glutathione peroxidase-1/2, and catalase were significantly elevated [140]. The observed changes were possibly due to downregulation of NF-ĸB pathway. Dimethyl fumarate has been reported to inhibit metastasis in cutaneous T cell lymphoma and melanoma through NF-ĸB pathway inhibition as well [139].

Curcumin is a natural compound found in Curcuma longa, possessing a variety of pharmacological properties, including antidiabetic, neuroprotective, anticancer, and anti-inflammation [141]. In NSCLC, migratory and invasive ability of A549 cells is reduced by curcumin through inhibition of adiponectin, an acid peptide hormone, via the NF-ĸB pathway [142]. Zerumbone, a monocyclic sesquiterpene compound, found in Zingiber zerumbet rhizomes has a broad range of pharmacological activities and anti-inflammatory effects. Recently, zerumbone was shown to suppress LPS-Induced inflammation in macrophages through inhibition of NLRP3 inflammasome. Furthermore, NF-ĸB activity and production of inflammatory cytokines, such as IL-1β and IL-6, were also significantly reduced [143]. The same inhibitory effect could also be seen in TNF-α-activated fibroblasts treated with zerumbone, in which tumor-promoting cytokines TNF-α, TGF-β, IL-33, SDF-1, and MCP-1 were significantly reduced in comparison to TNF-α-activated fibroblasts [144]. Astaxanthin, a naturally occurring xanthophyll carotenoid is found in marine organisms, such as algae, shrimp, and salmon [145]. Asthaxanthin possesses both anti-inflammatory and anti-oxidant properties and is shown to be capable of protecting the lung against inflammatory-based diseases. This compound exerts these effects by regulating nuclear factor erythroid 2-related factor/heme oxygenase-1, NF-ĸB, MAPK, JAK-STAT3, PI3-kinase/Akt pathways, and modulating immune response [145,146,147].

Farnesoid X receptor, known to regulate immune responses and inflammation in immune-mediated diseases, can promote tumor cell proliferation in NSCLC. Overexpression of this receptor in a Lewis lung carcinoma (LLC) syngeneic mouse model resulted in downregulation of PD-L1 [148]. The immunosuppressive role of farnesoid X receptor suggests it is a candidate for drug development. Additionally, it offers the opportunity for existing anti-PD-1 therapy to be fully utilized in treatment of NSCLC patients with high PD-1 expression. Bile acid and non-bile antagonists, such as Tauro-β-muricholic acid (T-β-MCA), taurochenodeoxycholic acid, glycoursodeoxycholic acid, guggulsterone, epiallopregnanolone sulfate, 3,5-disubstituted oxadiazole core, stigmasterol, tuberatolides, and andrographolide, are known to inhibit farnesoid X receptor [72]. These versatile natural compounds offer an alternative approach to curb cancer progression. Table 4 provides a list of natural compounds possessing anti-inflammation properties for NSCLC treatment.

## 6. Repurposing Drugs with Anti-Inflammation Properties and Their Delivery via Inhalation in NSCLC

Despite advances in treatment options, NSCLC’s mortality rates are still at an alarming state, and hence, a more precise and target-specific treatment is urgently needed to overcome this problem. Chronic inflammation plays an inevitable role in tumor initiation and progression. Thus, targeting inflammation presents an important tool in NSCLC treatment. There are various anti-inflammatory drugs and compounds that are readily available, which can be repurposed for NSCLC treatment.

Drug repurposing, also known as drug repositioning, is a process where the therapeutic use of an old or existing drug is explored for treatment of other indications. This is a highly efficient strategy with minimum risk of failure [152]. Another significant advantage of drug repurposing is that these drug candidates have already been clinically proven to be safe. Therefore, repurposed drugs are cost- and time-efficient as the pharmacokinetics and drug safety profile of the approved drugs have been fully investigated [153,154]. Drug repurposing is, hence, a promising option in targeting inflammation in NSCLC. Many compounds derived from natural products have shown promising anti-inflammatory activity and exhibit anticancer activity in NSCLC in pre-clinical studies. However, most suffer from a poor pharmacokinetic profile, which restricts further development.

Though many drugs have been shown to possess anti-inflammatory properties, there is a still a need to improve delivery of these drugs to target site. Localization of drugs at the intended site is vital to avoid toxicity issues related to their original indication. Statins, indicated to treat hypercholesterolemia, have low bioavailability, high first-pass metabolism in liver and intestine, rapid systemic clearance, and low half-life when administered orally. To overcome this issue, a high oral dose is, thus, required, which induces hepatotoxicity, rhabdomyolysis, nephrotoxicity, myalgia, and multiple drug interactions with other drugs and food [108]. Furthermore, NSAIDs can induce severe side effects in the cardiovascular, gastrointestinal, and renal systems, resulting in system failure in severe cases [155]. Thus, localized therapy provides an option to overcome these drawbacks.

Inhalation therapy, a localized therapy, directly delivers drug to the lung and minimizes systemic exposure and toxicity. Inhalation therapy provides rapid clinical response due to the ability to bypass therapeutic barriers, such as gastrointestinal absorption and first-pass liver metabolism [156]. The large surface area of lungs, highly dispersible nature of aerosol, and good epithelial permeability allow small molecules to be deposited in lungs and absorbed into circulation. Drug-metabolizing enzymes are lower in concentrations in lungs compared to the gastrointestinal tract and liver, thus allowing inhaled molecules to remain longer [157]. In addition, inhalation therapy can achieve either an equivalent or better therapeutic effect at a fraction of the systemic dose. An oral dose of 2–4 mg of salbutamol has been found to be equivalent to 100–200 μg by inhalation therapeutically [156]. Therefore, pulmonary drug administration is a promising route of drug delivery to improve the clinical efficacy of drug administered and minimizes unnecessary side-effects.

The administration of repurposed anti-inflammatory drugs for the treatment of NSCLC is one of the major challenges in achieving therapeutic efficacy. The non-localized administration of repurposed drug may cause other severe side effects, such as systemic toxicity and other toxicities which might be related to their original indications. To illustrate that, simvastatin, a type of drug indicated for the treatment of hyperlipidemia, exhibited a hypolipidemic effect when administered orally for cancer treatment, as it tends to accumulate in the liver [158]. Therefore, non-localized administration of repurposed drug cause unwanted effects, such as potential accumulation in organs such as the liver, spleen, and kidney. As a result, this causes failure of the repurposed drug in suppressing tumor growth in a more effective way. When dosage and frequency of drug administration was increased to overcome the above-mentioned disadvantage, there was the occurrence of multi-drug resistance and increased side effects. In addition to that, non-localized administration of the repurposed drug may also result in tumor recurrence [159]. Apart from that, naproxen was repurposed to treat bladder cancer and resulted in severe side effects, such as gastrointestinal, cardiovascular, and renal complications [160]. Thus, in order to specifically target NSCLC, localized therapy with inhalation therapy would help in improving the therapeutic efficacy and also in reducing unwanted systemic toxicity.

Various physicochemical properties influence drug deposition in various regions of the lung. Particle size, pKa, shape, lipophilicity (log P), and solubility are key factors moderating successful drug delivery to the lung [160]. Among these, particle size is a primary factor that influences particle deposition due to diminishing diameter of the airway towards the alveoli. Particles with larger molecular sizes have poorer ability to cross the air–blood barrier. Large drug molecules, however, possess a high receptor binding affinity, which can help reduce off-target effects [161]. Large molecular size drugs with sizes ranging from 1–3 μm are efficiently deposited in the lungs [162]. However, the effect of particle size can also be adjusted by incorporation of polymers. Nanoparticles with a diameter less than 200 nm, coated with polyethylene glycol (PEG), were found to be moving rapidly within mucus mesh. PEG-coated nanoparticles with a diameter larger than 500 nm were found almost immobilized in mucus mesh. Conversely, particle size can influence particles to escape clearance by macrophages where macrophages usually opsonized larger particles, with sizes ranging from 0.5 to 5 μm [163].

The rate and extent of drug uptake by lungs are altered by drug lipophilicity (log P) and pKa as the drug passes through surfactant, alveolar macrophage, mucus, and mucociliary clearance. Lipophilicity of drug impacts residence time of drug on surface of airways and influences therapeutic efficacy of the drug. Lipophilic drugs, having slower dissolution rates, undergo slower mucociliary clearance [164]. For example, inhalable corticosteroids have shown longer residence time in the lungs due to their slow dissolution rate and high lipophilicity [160]. Furthermore, drug delivery via inhalation is regulated by the morphology of drug particles. The aerodynamic performance of particles is influenced by particle shape, which affects their deposition. Particles with amorphous shape exhibit greater lung permeability compared to needle (crystalline)-shaped drug particle when administered through inhalation. In addition, pollen-shaped drug particles showed elevated lung deposition compared to drug particles of other shapes with the same aerodynamic size range [165].

Nanoformulation approaches for direct pulmonary delivery of repurposed drugs have extensively evolved since its first introduction in 1960s. In particular, lipid nanoparticles are important biodegradable and biocompatible delivery systems offering higher drug entrapment and site-specific delivery [166]. Nanoemulsions or microemulsions are thermodynamically stable oil-in-water emulsions and optically isotropic and transparent single-phase liquid suspensions. Microemulsions, such as Sandimmun Optoral and Neoral pre-concentrates, have shown superior solubilizing capacities compared to micellar solutions [167]. Nanoemulsions sizes range between 100 to 500 nm and are now commonly used as drug carriers in lipophilic active ingredients. Etomidat Lipuro, Diazepam Lipuro, Disoprivan, and Stesolid are among the current pharmaceutical nanoemulsions in the market [168]. Advantages of nanoemulsions over microemulsions are reduced local and systemic side effects, lesser pain during injection, and reduced hemolytic events.

Liposomes are spherical vesicles with membranes comprising one or more phospholipid (amphiphilic) bilayers, which are separated by an aqueous compartment. In the design of liposomes as drug carriers, natural phospholipids are often selected for their biological inertness, weak immunogenicity, and low intrinsic toxicity. Liposomes are classified based on vesicle size, lamellarity, and preparation. Unilamellar vesicles comprise one lipid bilayer with diameters between 50 to 500 nm, while multi-lamellar vesicles are envisioned as an onion skin-like arrangement comprising several concentric lipid bilayers with diameters between 1 to 5 µm. The multilamellar vesicles have high lipid content and can passively entrap drugs [169].

Using drug-loaded liposomes as delivery systems, drug concentrations at sites of action are seven- to nine-fold higher than using drugs alone without carriers [170]. The fact that liposomes may be administered parenterally renders it a good candidate as inhalable cargo to achieve an effective pulmonary drug delivery [160]. Currently, the downside of liposomes is that they are susceptible to removal by a mononuclear phagocytic system through non-specific binding of nanoparticle opsonizing serum proteins [171].

## 7. Conclusions

Comprehensive genomic profiling has allowed a more precise and personalized approach to treatment through tumor mutation profiles and PD-L1 expression in advanced NSCLC patients. However, lung cancer remains a leading cause of cancer-related deaths, despite the development and implementation of targeted therapy and immunotherapy in NSCLC. Inflammation exerts extensive roles within the lung TME to promote the development and progression of NSCLC. Repurposing of existing drugs and natural compounds exhibiting anti-inflammation properties provides an alternative paradigm in NSCLC therapy. Besides, repurposed anti-inflammatory drugs also provides a therapeutic option for NSCLC patients at an affordable cost in a short period of time. Repurposed drugs are time and cost effectiveness, as these drugs are already established on the market with regulatory approval. This reduces the time frame and money investment for the development of these repurposed drugs. However, most of these drugs are administered orally and are limited by their low bioavailability in the system to exert the desired effects. Localized therapy, such as pulmonary drug administration, tends to increase the drug deposition at the targeted organ with reduced unwanted toxicity and increased therapeutic effect. This requires the formulation of repurposed drugs to allow direct delivery to the lungs.

For a drug particle/molecule to diffuse through the extracellular matrices and reach the target organ and cells, it must possess favorable properties, such as small molecular weight, ideal lipophilicity, good solubility, and appropriate pKa, among others [160]. However, these desirable parameters have always been referred to as non-existent due to the highly complex interplay between molecule structure and pharmacodynamic/pharmacokinetic profiles. These requirements have severely restricted commercialization of many drugs for lung delivery. Although the ideal parameters could not fully be attained, they can still contribute to the overall effectiveness of a drug. Hence, an overall understanding to the challenges involved in drug distribution to reach the pulmonary site is highly necessary to ensure the success of drug repurposing and its effectiveness.

## Figures and Tables

**Table 1:** List of common targets of inflammation in NSCLC.

Targets	Role in Lung Cancer Inflammation	References
**Inflammatory cells**		
**Neutrophils**	Release proteases which degrade Tsp-1 and promotes tumor metastasis	[40]
**Macrophages**	Generate reactive oxygen and nitrogen intermediates, which induces DNA damage in proliferating cells, leading to neoplastic transformation	[15,21,33]
**Myeloid derived suppressor cells (MDSC)**	Degrade L-arginine, produce ROS, and secrete anti-inflammatory cytokines, such as IL-10 and TGF-β, to suppress the activity of other immune cells	[29,30]
**Gamma-delta (γδ) T cells**	Produce and release IL-17 and other effector molecules, which promotes inflammation and tumor proliferation	[44]
**Fibroblasts**	Produce and release inflammatory cytokines, such as MCP-1 and IL-6, in the tumor microenvironment	[37]
**Inflammatory cytokines**		
**IL-1β**	Increased IL-1β expression is linked to aggressive tumor biology and tumor invasiveness	[66]
**IL-4**		
**IL-6**	Produced by macrophages, T-lymphocytes, B-lymphocytes, and monocytes and promotes tumor cell proliferation, angiogenesis invasion, and metastasis	[67]
**IL-8**	Produced by endothelial cells, epithelial cells, and fibroblasts to promote angiogenesis, proliferation, and cancer cell invasion	[67]
**IL-9**	Through its effects on tumor-infiltrating T cells and tumor cell survival, promotes immune escape of lung tumor cells	[68]
**IL-13**	IL-13 has been linked to lung cancer metastasis and progression	[69]
**IL-17C**	Promotes tumorigenesis in Kras-driven lung cancer by inducing inflammation	[70]
**CCL5**	CCL5 production changes the immune microenvironment and encourages tumor growth	[71]
**HIF-1α**	Key mediator of adaptation to hypoxic condition and promotes tumorigenesis via inflammation	[25]
**TNF-α**	Tumor necrosis factor-alpha (TNF-α) controls inflammation and tumor development in non-small cell lung cancer (NSCLC)	[72]
**Inflammatory gene expressions**		
**ISOC1**	Participates in DNA damage repair and inflammation to promote lung cancer development	[73]
**Ezh2**	Ezh2 inhibition amplifies inflammation in Kras-driven lung cancer	[74]
**LRRK2**	Loss of LRRK2 promotes tumor initiation and size (tumorigenesis)	[75]
**Signaling Proteins**		
**NF-ĸB**	Promotes tumor formation by inducing inflammation	[76]
**JAK/STAT3**		[36]
**JNK1**		[76]

**Table 2:** List of existing drugs with anti-inflammation properties for NSCLC treatment.

Drug	Mechanism of Action	Initial Purpose	Performance Remarks	Reference
**Statins** - **Simvastatin** - **Pravastatin** - **Rosuvastatin** - **Pitavastatin** - **Atorvastatin**	Inhibits 3-hydroxy-3-methylglutaryl coenzyme A (HMG CoA) reductase	To treat hypercholesterolemia	In vivo—Atorvastatin showed better anti-inflammatory properties than simvastatin	[108]
**Thioridazine**	Inhibits IκBα protein degradation, NF-ĸB activation	Anti-psychotic drug against schizophrenia	In vivo—potent anti-inflammatory target specific drug	[87]
**Thalidomide**	Inhibits the production of pro-inflammatory cytokines (TNF-α, IL-1α)	To treat morning sickness in pregnant women	In vivo—significant reduction in pro-inflammatory cytokines in pneumonia-induced acute lung inflammation	[109]
**Fingolimod (FTY720)**	Inhibits SphK/S1P signaling and S1PR3 in lung cancer metastasis	To treat multiple sclerosis		[110]
**Anastrozole**	In combination with non-steroidal anti-inflammatory drug (Aspirin) to reduce circulating Beta-estradiol, pro-inflammatory cytokines, and macrophages recruitment in a tobacco induced lung cancer model	Hormone therapy	In vivo—downregulation of SOX-2 expression in the lungs	[102]
**NSAIDS** - **Aspirin** - **Aspirin/Naproxen** - **Sulindac Acid** - **Amino salicylic acid** - **Celecoxib**	Downregulate COX-2 expression Upregulation of bax expression and downregulation of bcl-2 expression Degrade antiapoptotic protein bcl-XL Increase ROS Increase p53 gene expression	To treat inflammation, antipyretic, analgesics		[111]
**Tyrosine Kinase Inhibitor** - **Imatinib mesylate**	Inhibits LPS-induced production of TNF-a, IL-6, and IL-8, via inhibition of nuclear factor kappa B (NF-ĸB)	To treat leukemias characterized by the presence of the Philadelphia chromosome. Recently, it has been proposed to treat inflammation linked to COVID-19 infection	Significant decrease of NF-ĸB in chronic myelogenous leukemia patients	[88,90]
**H1 histamine antagonist** - **Clemastine**	Reduces NF-ĸB activity and TLR4 expression	To treat allergy symptoms		[89]
**Phosphodiesterase Inhibitor** - **Ibudilast**	Inhibit NF-ĸB by preventing nuclear translocation	To treat asthma and stroke		[89]
**Interleukin-6 inhibitors** - **Sarilumab** - **Tocilizumab** - **Siltuximab**	Monocolonal antibodies inhibit IL-6 receptor and IL-6	Treatment of inflammatory diseases such as rheumatoid arthritis and COVID-19 infection		[95]
**Interleukin-1 inhibitors** - **Anakinra** - **Rilonacept** - **Canakinumab**	Inhibits IL-1 directly or binds to IL-1 receptor			[112]
**HIF-1α inhibitors** - **2ME2 NCD (panzem)** - **17-AAG (tanespimycin)** - **Vorinostat (SAHA, Zolinza)** - **PT2385** - **PT2977** - **EZN-2208 (Pegylated SN-38)** - **CRLX101**	Inhibits HIF-1α by either inhibiting its production, promoting the degradation, interfering the signaling pathway, or direct binding			[31]

**Table 3:** Drugs currently undergoing clinical trials for the treatment of non-small cell lung cancer.

Trial Number	Phase	Status	Estimated Completion Date	Treatment
**NCT04648033**	1	Recruiting	December 2027	Vancomycin + Stereotactic Body Radiation Therapy
**NCT04905316**	2	Recruiting	May 2024	Canakinumab + Durvalumab + Radiation therapy + Chemotherapy
**NCT04382300**	2	Recruiting	April 2023	Pyrotinib + thalidomide
**NCT02779751**	1	Active, not recruiting	September 2023	Abemaciclib + Pembrolizumab + Anastrozole
**NCT04184921**	-	Active, not recruiting	December 2023	Aspirin + Osimertinib
**NCT00408460**	2	Completed	April 2017	Imatinib Mesylate + paclitaxel
**NCT05704634**	1	Not yet recruiting	January 2028	Cemiplimab + Sarilumab
**NCT04691817**	2	Not yet recruiting	April-2026	Atezolizumab + Tocilizumab
**NCT03337698**	1 and 2	Recruiting	August 2025	Atezolizumab + Cobimetinib + RO6958688 + Docetaxel + CPI-444 + Pemetrexed + Carboplatin + Gemcitabine + Linagliptin + Tocilizumab + Ipatasertib + Bevacizumab + Sacituzumab Govitecan + Radiation + Evolocumab
**NCT02638090**	1 and 2	Active, not recruiting	January 2024	Vorinostat + Pembrolizumab
**NCT01380769**	2	Completed	February 2014	CRLX101
**NCT05636592**	1	Recruiting	December 2027	Statins + PD-1/PD-L1 inhibitors
**NCT05445791**	3	Recruiting	July 2025	Metformin hydrochloride

**Table 4:** List of natural compounds to target inflammation in NSCLC.

Compound	Mechanism of Action	Initial Purpose	Performance Remarks	Reference
**Andrographolide**	Inhibition of NF-ĸB	Treatment of upper airway disorders		[122,124,125]
**Asthaxanthin**	Regulating the nuclear factor erythroid 2-related factor/heme oxygenase-1 pathway, NF-ĸB signaling, MAPK signaling, JAK-STAT 3 signaling, Pi3-kinase/Akt pathway, and modulating immune response	Dietary supplement		[145,146,147]
**Curcumin**	Inhibition of NF-ĸB	Dietary supplement		[149,150]
**Fumaric Acid Esters**	Alters leukocyte, keratinocyte, and/or endothelial functions	To treat psoriasis and multiple sclerosis		[139]
**Baicalein**	Inhibits metastasis (exact mechanism of action yet to be confirmed)			[136]
**Kampo medicine, Hochuekkito, TJ-41**	Inhibited influenza A virus replication by IFN-α upregulation	To treat infectious disease, possesses virological activity	In vivo and in vitro study shows positive results	[117]
**Cinnamon** **(cinnamaldehyde, cinnamic acid, 2-hydroxycinnamaldehyde, 2-methoxycinnamaldehyde, and eugenol)**	Suppressed nitric oxide (NO), IL-6, TNF-α, and IL-1β production. Production and blocking of nuclear factor-ĸB (NF-ĸB) and mitogen-activated protein kinase (MAPK)	Has immunomodulator, antiseptic and antiviral properties		[114]
**β-carbolines**	Inhibits NF-κB/p65 and EMT transition	To treat altitude sickness and possess anti-inflammatory properties		[151]
**Magnesium isoglycyrrhizinate (MgIG)**	Inhibits fibroblast differentiation via the p38MAPK/Nox4/Akt pathway	Respiratory disorders, hyperdipsia, epilepsy, fever		[151]
**Polydatin (PD)**	NLRP3 inflammasome and NF-κB pathway	Used to reduce symptoms of menopause, digestive system		[151]
**Zingerone (vanillylacetone)**	Inhibiting NF-κB and MAPKs	To treat infections, nausea, bronchitis, dysentery, heartburn, cough, flatulence, diarrhea, loss of appetite		[151]
**Zerumbone**	Inhibits TNF-α or LPS-induced production inflammatory cytokines via inhibition of NF-ĸB	To treat fever, sprains, asthma, torment, severe sprains, toothache, allergies, wounds, and stomachache		[143]

## Data Availability

Not applicable.
